# Parasites (Monogenea) of tilapias *Oreochromis niloticus* and *Coptodon rendalli* (Cichlidae) in a river spring in Brazil[Fn FN1]

**DOI:** 10.1051/parasite/2024021

**Published:** 2024-04-10

**Authors:** Mariana Bertholdi Ebert, Rodrigo Bravin Narciso, Diego Henrique Mirandola Vieira Dias, Melissa Miyuki Osaki-Pereira, Maurício Jorge, Gerardo Pérez-Ponce de León, Reinaldo José da Silva

**Affiliations:** 1 São Paulo State University (UNESP), Institute of Biosciences, Department of Biodiversity and Biostatistics, Section of Parasitology Botucatu SP Brazil; 2 Escuela Nacional de Estudios Superiores, Unidad Mérida, Universidad Nacional Autónoma de México 97357 Ucú Yucatán Mexico

**Keywords:** Monogenea, *Cichlidogyrus*, *Scutogyrus*, Neotropical region, LSU rDNA, Exotic fishes

## Abstract

In the present study, we examined 30 individuals of introduced African cichlids, *Oreochromis niloticus* and *Coptodon rendalli*, collected in a river spring of the Pardo River, Paranapanema River basin, southeastern Brazil. Based on morphological and molecular analyses of the partial LSU rDNA gene, we identified four species of monogeneans, *Cichlidogyrus tilapiae*, *C. thurstonae*, *C. mbirizei,* and *Scutogyrus longicornis* on the gills of *O. niloticus*, whereas individuals of *C. rendalli* were infested only with *C. papernastrema*. This is the first record of *C. mbirizei* and *C. papernastrema* in tilapias from Brazil. The ecological consequences of the introduction of exotic species of tilapia such as *O. niloticus* and *C. rendalli* along with their monogenean parasites in a wild environment represented by a river spring are discussed. Our new molecular data on *Cichlidogyrus* and *Scutogyrus* contribute to the investigation of the phylogenetic interrelationships of these widely distributed genera of monogeneans since their species composition is still unsettled.

## Abstract (fr)

Dans la présente étude, nous avons examiné 30 individus de cichlidés africains introduits, *Oreochromis niloticus* et *Coptodon rendalli*, collectés dans une source fluviale du fleuve Pardo, bassin du fleuve Paranapanema, dans le sud-est du Brésil. Sur la base d’analyses morphologiques et moléculaires du gène partiel de l’ADNr LSU, nous avons identifié quatre espèces de monogènes, *Cichlidogyrus tilapiae*, *C. thurstonae*, *C. mbirizei* et *Scutogyrus longicornis* sur les branchies d’*O. niloticus*, alors que les individus de *C. rendalli* étaient infestés uniquement par *C. papernastrema*. Il s’agit du premier signalement de *C. mbirizei* et *C. papernastrema* chez des tilapias du Brésil. Les conséquences écologiques de l’introduction d’espèces exotiques de tilapia telles que *O. niloticus* et *C. rendalli* ainsi que leurs monogènes parasites dans un environnement sauvage représenté par une source fluviale sont discutées. Nos nouvelles données moléculaires sur *Cichlidogyrus* et *Scutogyrus* contribuent à l’étude des interrelations phylogénétiques de ces genres de monogènes largement distribués puisque leur composition spécifique est encore incertaine.

## Introduction

Cichlids belonging to genera *Oreochromis* Günther, 1889 and *Coptodon* Gervais, 1853, commonly known as tilapias, are subtropical to tropical freshwater fishes that are native to Africa and the southwestern Middle East [11]. Over the past nine decades, tilapias have been intentionally dispersed worldwide to be used as biological control of aquatic weeds and insects, as baitfish in fisheries, as aquarium species, and as a source of food protein [11].

Tilapias have been translocated outside their native range and currently they are farmed in 79 countries; tilapia production in countries such as China, Indonesia and Bangladesh accounts for 66.76% of all tilapia grown [68]. In Brazil, two tilapia species are commonly found primarily on fish farms, although they have been fortuitously released to wild environments. The red-breasted tilapia, *Coptodon rendalli* (Boulenger, 1897) (= *Chromis rendalli* Boulenger, 1897; *Tilapia rendalli* (Boulenger, 1897)) was the first to be introduced to the country in the 1950s with the purpose of populating dams of electric energy companies in São Paulo state [13]; and a while later, the Nile tilapia *Oreochromis niloticus* (Linnaeus, 1758) (= *Perca nilotica* Linnaeus, 1758) was introduced as part of a federal government program against hunger [13]. Since then, the two species have been intensely farmed on fish farms and “fish and pay” establishments around the country, to promote developing economies and recreation [6, 7]. Even though Brazil is one of the world’s richest in the diversity of native ichthyofauna [64], tilapias are presently the main commercially cultivated species in national aquaculture, and the most consumed fishes in the country [13, 55, 74].

Since their introduction for aquacultural purposes, *C. rendalli* and *O. niloticus* have been intentionally and accidentally spread into several lakes, dams, and reservoirs [2, 54], and also into the basins of several rivers throughout the country, namely: Tietê [54], Paraná [9], Doce [72], Paranapanema [12, 44], Grande [79], Itajaí [23, 29], Uruguay [46], and even the Amazon [5, 55, 73]. The presence of non-native species such as *C. rendalli* and *O. niloticus* is a major threat to local biodiversity [70] since they can affect native biota not only through direct interactions, *e.g.*, competition [15] but also through indirect interactions such as parasites which are usually co-introduced with their hosts [71]. A serious problem of co-introducing parasites is that they might switch hosts and successfully infect native species [24], increasing the risk of decline and/or extinction of native fauna [24, 71].

African cichlid fishes are known to harbour species contained in six genera of monogenean parasites; two of them are mesoparasites (*i.e.*, *Enterogyrus* Paperna, 1963 and *Urogyrus* Bilong Bilong, Birgi & Euzet, 1994) which live in a host’s body cavity, and four are ectoparasites found on the gills or the body surface [21, 56] feeding on mucus, skin and possibly the blood of their hosts [25]. The monogenean gill parasites include three genera belonging to the Dactylogyridae Bychowsky, 1933, *i.e.*, *Cichlidogyrus* Paperna, 1960, *Onchobdella* Paperna, 1968, and *Scutogyrus* Pariselle & Euzet, 1995, and one genus belonging to the Gyrodactylidae Cobbold, 1864, *i.e.*, *Gyrodactylus* von Nordmann, 1832 [21, 56]. *Cichlidogyrus* is one of the most diverse genera of monogeneans parasitizing cichlid fishes and is found in more than 40 fish species across 11 different genera [60]. To date, 130 species of *Cichlidogyrus* are considered valid [76]. In contrast, *Scutogyrus* is presently represented by only seven species and known to be restricted to cichlids of the genera *Sarotherodon* Rüppell, 1852 and *Oreochromis* [43, 69], although *S. longicornis* has been reported parasitizing *C. rendalli* in Brazil [24, 77].

Among other monogenean parasites (see [18, 55, 68, 72]), species of *Cichlidogyrus* and *Scutogyrus* have been commonly reported parasitizing tilapias in Brazil: *O. niloticus* was found infested with *Cichlidogyrus tilapiae* Paperna, 1960 [4, 8, 19, 41, 49], *Cichlidogyrus halli* (Price & Kirk, 1967) (= *Cleidodiscus halli* Price & Kirk, 1967) [8, 18, 29, 34, 79], *Cichlidogyrus thurstonae* Ergens, 1981 [8, 18, 23, 29, 34, 46, 79] *Cichlidogyrus sclerosus* [3, 8, 18, 23, 26, 29, 34, 44, 45, 69], *Cichlidogyrus rognoni* Pariselle, Bilong Bilong & Euzet, 2003 [8, 18], and *Scutogyrus longicornis* (Paperna & Thurston, 1969) (= *Cichlidogyrus longicornis* Paperna & Thurston, 1969) [8, 18, 29, 34, 45, 79]. As for *C. rendalli*, only *C. tilapiae*, *Cichlidogyrus* sp. and *S. longicornis* were reported to parasitize this species in Brazil [26, 73]. These monogeneans are native to the African continent [56] and the presence of these species in wild populations in the freshwaters of Brazil is strong evidence of their co-introduction into the country along with their fish hosts.

This study aimed to report the monogenean parasites found on the gills of the introduced cichlid fishes *C. rendalli* and *O. niloticus* from the Pardo River, Paranapanema River basin, São Paulo state, Brazil. The morphology and phylogenetic relationships using the LSU rDNA gene of four species of *Cichlidogyrus* and one species of *Scutogyrus* found on the gills of the fishes were investigated. The ecological consequences of the presence of exotic species such as *C. rendalli* and *O. niloticus* in a river spring are also discussed.

## Material and methods

### Host sampling, parasitological procedures and ethics

During an inventory of fishes and parasites in the Pardo River, Paranapanema River basin, São Paulo state, Brazil, 20 specimens of red-breasted tilapia *C. rendalli* and 10 specimens of Nile tilapia *O. niloticus* were collected near the river spring (23°0′19.00″ S; 48°22′34.25″ W), municipality of Botucatu, in June 2021. Tilapias were introduced into the region by farm owners who, without proper technical knowledge, attempted to produce fishes for commercial purposes or other reasons.

The fishes were collected using casting nets and euthanized with sodium thiopental (Thiopentax^®^). The specimens were then individually stored in plastic bags and frozen to later conduct necropsy at the laboratory, while others were examined *in situ* to collect fresh monogeneans, which were placed into 96% molecular-grade ethanol for molecular analyses. At the laboratory, gills were removed, placed in Petri dishes with tap water, and analyzed for monogeneans under a stereomicroscope. Monogeneans were counted, separated from the gills, and mounted on slides with Hoyer and Gray and Wess’ medium [41].

The morphology of the sclerotized structures of the haptor (bar, anchors, and hooks) and copulatory complex of the monogeneans was analyzed using a V3 Leica Application Suite (LAS) computerized system for image analysis adapted to a microscope with differential interference contrast. Measurements of the specimens (total body length, haptoral characters (bars, anchors, and hooks), heel, penis, accessory piece of the male copulatory organ, and vagina) were made following Euzet & Prost [16] and Jorissen *et al*. [32]. Ecological data such as the prevalence, abundance, and mean intensity of infestation, were calculated following Bush *et al*. [10].

Representative specimens of the monogeneans were deposited in the Helminthological Collection of the Institute of Biosciences (CHIBB), Botucatu, São Paulo state, Brazil, under numbers: *S. longicornis* 740L; *C. mbirizei* 741L -744L; *C. thurstonae* 745L-749L; *C. tilapiae* 750L-754L; *C. papernastrema* 755L-759L. Representative fish hosts were deposited in the Fish Collection of the Laboratory of Fish Biology and Genetics (LBP) at the São Paulo State University (UNESP), Botucatu, Brazil, under numbers 33636–33638. Fishes were collected under the authorization of the Instituto Chico Mendes de Conservação da Biodiversidade (SISBIO #60640-1). All procedures followed the recommendations and approval of the Ethics Committee for Animal Experimentation of the São Paulo State University (UNESP), Institute of Biosciences, Botucatu, Brazil (CEUA 9415260520). According to Brazilian laws, species registration for scientific research purposes was carried out at SisGen (AD05367).

### Molecular and phylogenetic analyses

Genomic DNA of the monogeneans was extracted using a DNeasy Blood & Tissue Kit (QIAGEN, Valencia, CA, USA), following the manufacturer’s protocol. Fragments of the LSU rDNA gene were amplified using the primers 382F (5′-AGCTGGTGGAGTCAAGCTTC-3′) and 1289R (5′-TGCTCACGTTTGACGATCGA-3′) [17], using the following cycling conditions: initial denaturation of 5 min at 95 °C followed by 40 cycles of 95 °C for 30 s, 56 °C for 30 s, 72 °C for 2 min, and a final extension of 10 min at 72 °C [17]. Conventional polymerase chain reaction (PCR) amplifications were performed on a final volume of 25 μL containing 12.5 μL of 2× MyFi^TM^ Mix (Bioline, Taunton, MA, USA), 3.0 μL of extracted DNA, 7.5 μL of pure water and 1.0 μL of each PCR primer. PCR products (2.0 μL) were run on an agarose gel (1%) using GelRed^TM^ fluorescent nucleic acid dye and loading buffer to confirm amplicon size and yield. PCR amplicons were purified using the QIAquick PCR Purification Kit (QIAGEN), following the manufacturer’s instructions. Automated sequencing was performed directly on purified PCR products using a BigDye v.3.1 Terminator Cycle Sequencing Ready Reaction kit on an ABI 3500 DNA genetic sequencer (Applied Biosystems, Waltham, MA, USA). Forward and reverse sequences were assembled and edited using Sequencher v. 5.2.4 (Gene Codes, Ann Arbor, MI, USA).

To investigate the phylogenetic position of our newly generated partial LSU rDNA sequences, a dataset was created and complemented with published sequences of *Cichlidogyrus* spp. and *Scutogyrus* spp. retrieved from GenBank (Table 1). *Cichlidogyrus pouyaudi* Pariselle & Euzet, 1994, *Cichlidogyrus berrebi* Pariselle & Euzet, 1994 and *Cichlidogyrus kothiasi* Pariselle & Euzet, 1994 were used as outgroups because of their basal position assumed in the phylogenetic tree delivered by Mendlová *et al.* [49], Cruz-Laufer *et al.* [14]. Alignment of the dataset was performed using the MUSCLE algorithm implemented on Geneious 7.1.3 [36] with default settings. The best-fitting model of nucleotide substitution for the aligned dataset was selected in the JModelTest software [58] using the Akaike information criterion, as GTR+I+G.

**Table 1:** List of monogeneans included in the phylogenetic analyses, with details of the host, locality, GenBank accession numbers of sequences from the LSU rDNA gene, and their references. New sequences obtained for the present study are in bold.

Monogenea species	Host species	Locality	LSU accession numbers	References
**Dactylogyridae**				
*Cichlidogyrus acerbus* Dossou, 1982	*Sarotherodon galilaeus* (Linnaeus, 1758)	Senegal	HQ010036	[48]
*Cichlidogyrus aegypticus* Ergens, 1981	*Coptodon guineensis* (Günther, 1862)	Senegal	HQ010021	[48]
*Cichlidogyrus amieti* Birgi & Euzet, 1983	*Aphyosemion cameronense* (Boulenger, 1903)	Cameroon	KT945076	[51]
*Cichlidogyrus amphoratus* Pariselle & Euzet, 1996	*Coptodon guineensis*	Senegal	HE792772	[49]
*Cichlidogyrus arthracanthus* Paperna, 1960	*Coptodon guineensis*	Senegal	HQ010022	[48]
*Cichlidogyrus attenboroughi* Kmentová et al., 2016	*Benthochromis tricoti* (Poll, 1948)	Burundi	MH708146	[39]
*Cichlidogyrus berrebii* Pariselle & Euzet, 1994	*Tylochromis jentinki* (Steindachner, 1894)	Ivory Coast	MW580321	[14]
*Cichlidogyrus brunnensis* Kmentová et al., 2016	*Trematocara unimaculatum* Boulenger, 1901	Burundi	MH708144	[39]
*Cichlidogyrus buescheri* Pariselle & Vanhove, 2015	*Interochromis loocki* (Poll, 1949)	Zambia	MW580322	[14]
*Cichlidogyrus casuarinus* Pariselle et al., 2015	*Bathybates minor* Boulenger, 1905	Burundi	KX007796	[38]
*Cichlidogyrus centesimus* Vanhove, Volckaert & Pariselle, 2011	*Ophthalmotilapia ventralis* (Boulenger, 1898)	Congo	MW580328	[14]
*Cichlidogyrus cirratus* Paperna, 1964	*Oreochromis niloticus* (Linnaeus, 1758)	Senegal	HE792773	[49]
*Cichlidogyrus consobrini* Jorissen, Pariselle & Vanhove 2017	*Sargochromis mellandi* (Boulenger, 1905)	Congo	OM720080	[33]
*Cichlidogyrus cubitus* Dossou, 1982	*Coptodon guineensis*	Senegal	HQ010037	[48]
*Cichlidogyrus digitatus* Dossou, 1982	*Coptodon guineensis*	Senegal	HQ010023	[48]
*Cichlidogyrus dossoui* Douëllou, 1993	*Coptodon rendalli* (Boulenger, 1897)	Zambia	MW580335	[14]
*Cichlidogyrus douellouae* Pariselle, Bilong & Euzet, 2003	*Sarotherodon galilaeus*	Senegal	HE792774	[49]
*Cichlidogyrus dracolemma* Řehulková, Mendlová & Šimková, 2013	*Hemichromis letournaeuxi* Sauvage, 1880	Senegal	HQ010027	[48]
*Cichlidogyrus ergensi* Dossou, 1982	*Coptodon guineensis*	Senegal	HQ010038	[48]
*Cichlidogyrus falcifer* Dossou & Birgi, 1984	*Hemichromis fasciatus* Peters, 1857	Senegal	HQ010024	[48]
*Cichlidogyrus gillardinae* Muterezi et al., 2012	*Astatotilapia burtoni* (Günther, 1894)	Congo	MW580338	[14]
*Cichlidogyrus halli* (Price & Kirk, 1967)	*Sarotherodon galilaeus*	Senegal	HQ010025	[48]
*Cichlidogyrus halli*	*Oreochromis niloticus*	Madagascar	MH767403	[72]
*Cichlidogyrus irenae* Gillardin et al., 2012	*Gnathochromis pfefferi* (Boulenger, 1898)	Burundi	MH708145	[39]
*Cichlidogyrus kothiasi* Pariselle & Euzet, 1994	*Tylochromis jentinki* (Steindachner, 1894)	Ivory Coast	MW580341	[14]
*Cichlidogyrus longicirrus* Paperna, 1965	*Hemichromis fasciatus*	Senegal	HQ010026	[48]
*Cichlidogyrus mbirizei* Muterezi et al., 2012	*Oreochromis niloticus x mweruensis*	Congo	MG973076	[10]
*Cichlidogyrus nageus* Řehulková, Mendlová & Šimková, 2013	*Sarotherodon galilaeus*	Senegal	HQ010028	[48]
*Cichlidogyrus nandidae* Birgi & Lambert, 1986	*Polycentropsis abbreviata* (Boulenger, 1901)	Cameroon	MW580344	[14]
*Cichlidogyrus njinei* Pariselle, Bilong & Euzet, 2003	*Sarotherodon galilaeus*	Senegal	HE792775	[49]
*Cichlidogyrus nshomboi* Muterezi et al., 2012	*Boulengerochromis microlepis* (Boulenger, 1899)	Congo	MW580345	[14]
*Cichlidogyrus papernastrema* Price, Peebles & Bamford, 1969	*Coptodon rendalli*	Congo	MW580347	[14]
*Cichlidogyrus papernastrema*	*Tilapia sparrmanii* Smith, 1840	Congo	OM720075	[33]
*Cichlidogyrus papernastrema*	*Tilapia sparrmanii*	Congo	OM720076	[33]
***Cichlidogyrus papernastrema***	***Oreochromis niloticus***	**Brazil**	**PP477267**	**Present study**
***Cichlidogyrus papernastrema***	***Oreochromis niloticus***	**Brazil**	**PP477261**	**Present study**
***Cichlidogyrus papernastrema***	***Oreochromis niloticus***	**Brazil**	**PP477262**	**Present study**
***Cichlidogyrus papernastrema***	***Oreochromis niloticus***	**Brazil**	**PP477263**	**Present study**
***Cichlidogyrus papernastrema***	***Oreochromis niloticus***	**Brazil**	**PP477264**	**Present study**
***Cichlidogyrus papernastrema***	***Oreochromis niloticus***	**Brazil**	**PP477265**	**Present study**
***Cichlidogyrus papernastrema***	***Oreochromis niloticus***	**Brazil**	**PP477266**	**Present study**
***Cichlidogyrus papernastrema***	***Oreochromis niloticus***	**Brazil**	**PP477268**	**Present study**
***Cichlidogyrus papernastrema***	***Oreochromis niloticus***	**Brazil**	**PP477269**	**Present study**
*Cichlidogyrus philander* Douëllou, 1993	*Pseudocrenilabrus philander* (Weber, 1897)	South Africa	MG279691	[28]
*Cichlidogyrus pouyaudi* Pariselle & Euzet 1994	*Tylochromis intermedius* (Boulenger, 1916)	Senegal	HQ010039	[48]
*Cichlidogyrus quaestio* Douëllou, 1993	*Coptodon rendalli*	Congo	OM720059	[33]
*Cichlidogyrus sanseoi* Pariselle & Euzet 2004	*Hemichromis fasciatus*	Senegal	MW580348	[14]
*Cichlidogyrus schreyenbrichardorum* Pariselle & Vanhove, 2015	*Interochromis loocki*	Zambia	MW580349	[14]
*Cichlidogyrus sclerosus* Paperna & Thurston, 1969	*Oreochromis niloticus*	China	DQ157660	[11]
*Cichlidogyrus teugelsi* Pariselle & Euzet 2004	*Hemichromis fasciatus*	Ivory Coast	MW580354	[14]
*Cichlidogyrus thurstonae* Ergens, 1981	*Oreochromis niloticus*	Brazil	OM720063	[33]
***Cichlidogyrus thurstonae***	***Oreochromis niloticus***	**Brazil**	**PP477271**	**Present study**
*Cichlidogyrus thurstonae*	*Oreochromis niloticus*	Brazil	OM720064	[33]
*Cichlidogyrus thurstonae*	*Paretroplus lamenabe*	Brazil	MH767406	[72]
*Cichlidogyrus tiberianus* Paperna, 1960	*Coptodon guineensis*	Senegal	HE792776	[49]
*Cichlidogyrus tilapiae* Paperna, 1960	*Coptodon rendalli*	Madagascar	MH767407	[72]
***Cichlidogyrus tilapiae***	***Oreochromis niloticus***	**Brazil**	**PP477257**	**Present study**
***Cichlidogyrus tilapiae***	***Oreochromis niloticus***	**Brazil**	**PP477258**	**Present study**
***Cichlidogyrus tilapiae***	***Oreochromis niloticus***	**Brazil**	**PP477259**	**Present study**
***Cichlidogyrus tilapiae***	***Oreochromis niloticus***	**Brazil**	**PP477260**	**Present study**
*Cichlidogyrus tilapiae*	*Pachypanchax omalonotus*	Madagascar	MH767410	[72]
*Cichlidogyrus tilapiae*	*Oreochromis mweruensis* Trewavas, 1983	Congo	OM720054	[33]
*Cichlidogyrus tilapiae*	*Hemichromis fasciatus*	Senegal	HQ010029	[48]
*Cichlidogyrus vandekerkovei* Vanhove, Volckaert & Pariselle, 2011	*Ophthalmotilapia ventralis* Boulenger, 1898	Congo	MW580356	[14]
*Cichlidogyrus vealli* Pariselle & Vanhove, 2015	*Interochromis loocki*	Zambia	MW580358	[14]
*Cichlidogyrus yanni* Pariselle & Euzet, 1996	*Coptodon guineensis*	Senegal	HE792777	[49]
*Cichlidogyrus zambezensis* Douëllou, 1993	*Serranochromis macrocephalus* (Boulenger, 1899)	Congo	MW580361	[14]
*Scutogyrus bailloni* Pariselle & Euzet, 1995	*Sarotherodon galilaeus*	Ivory Coast	HE792778	[49]
*Scutogyrus gravivaginus* (Paperna & Thurston 1969)	*Oreochromis mweruensis*	Congo	OM720073	[33]
*Scutogyrus longicornis* (Paperna & Thurston 1969)	*Oreochromis niloticus*	Senegal	HQ010035	[49]
***Scutogyrus longicornis***	***Oreochromis niloticus***	**Brazil**	**PP477270**	**Present study**
*Scutogyrus minus* (Dossou, 1982)	*Sarotherodon melanotheron* Rüppel, 1852	Ivory Coast	HE792779	[49]
*Scutogyrus vanhovei* Pariselle et al., 2013	*Pelmatolapia mariae* Boulenger, 1899	Cameroon	MW580366	[14]

Phylogenetic trees were obtained using Bayesian Inference (BI) and Maximum Likelihood (ML). The BI analysis was performed using MrBayes 3.2 [66] on the online platform CIPRES [52]. The Markov chain Monte Carlo (MCMC) was run with 10^6^ generations saving one tree every 100 generations, with a burn-in set to the first 25% of the trees. Only nodes with posterior probabilities (pp) greater than 0.90 were considered well-supported. The ML analysis was run in RAxML [27] at the online platform CIPRES [52] with 1000 bootstrap replicates. Only nodes with bootstrap values greater than 70 were considered well-supported. The BI and ML trees were visualized in FigTree v. 1.3.1 software [62] and edited in CorelDRAW X6. Pairwise genetic distances among and between sequences were calculated using the Kimura-2-parameter (K2P) model and a bootstrap procedure with 1000 replicates in the MEGA11 program [37].

## Results

### Morphological analyses

**Figure 1:**
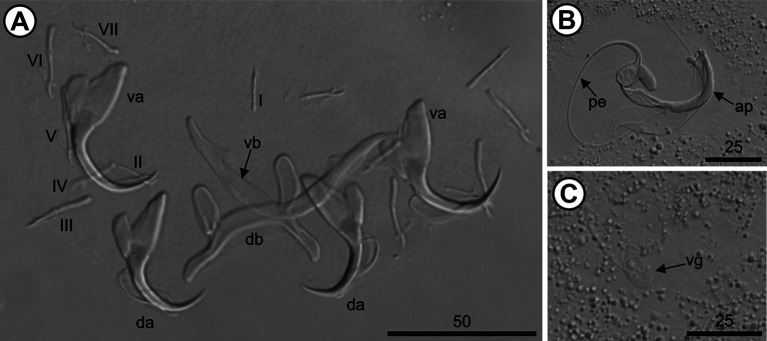
*Cichlidogyrus mbirizei* Muterezi Bukinga, Vanhove, Van Steenberge & Pariselle, 2012 from the gills of *Oreochromis niloticus* (Linnaeus, 1758) from Pardo River, Paranapanema River basin, São Paulo state, Brazil. A. Haptor; B. Male copulatory organ; C. Vagina. Hoyer’s mounting medium. Legend: ap, accessory piece; da, dorsal anchor, db, dorsal bar; I–VII, hook pairs; pe, penis; va, ventral anchor; vb, ventral bar; and vg, vagina.

**Figure 2:**
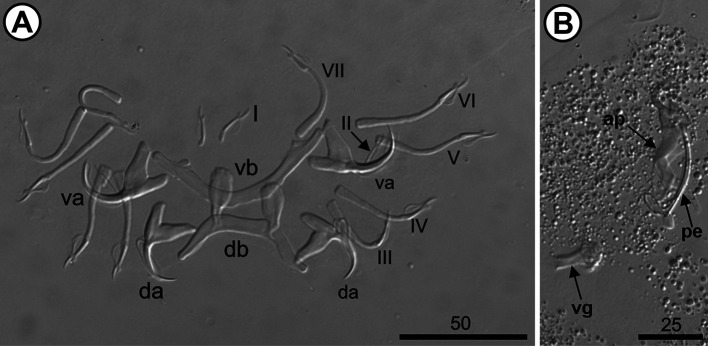
*Cichlidogyrus thurstonae* Ergens, 1981 from the gills of *Oreochromis niloticus* (Linnaeus, 1758) from Pardo River, Paranapanema River basin, São Paulo state, Brazil. A. Haptor; B. Male copulatory complex. Hoyer’s mounting medium. Legend: ap, accessory piece; da, dorsal anchor; db, dorsal bar; I–VII, hook pairs; pe, penis; va, ventral anchor; vb, ventral bar; and vg, vagina.

**Figure 3:**
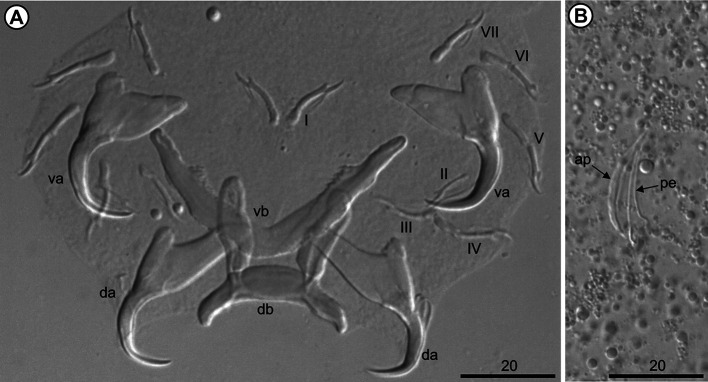
*Cichlidogyrus tilapiae* Paperna, 1960 from the gills of *Oreochromis niloticus* (Linnaeus, 1758) from Pardo River, Paranapanema River basin, São Paulo state, Brazil. A. Haptor; B. Male copulatory complex. Hoyer’s mounting medium. Legend: ap, accessory piece; da, dorsal anchor; db, dorsal bar; I–VII, hook pairs; pe, penis; va, ventral anchor; and vb, ventral bar.

**Figure 4:**
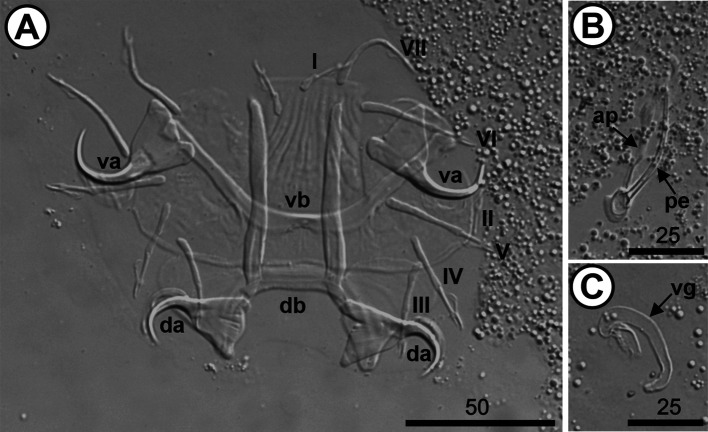
*Scutogyrus longicornis* (Paperna & Thurston, 1969) from the gills of *Oreochromis niloticus* (Linnaeus, 1758) from Pardo River, Paranapanema River basin, São Paulo state, Brazil. A. Haptor; B. Male copulatory complex; C. Vagina. Hoyer’s mounting medium. Legend: ap, accessory piece; da, dorsal anchor; db, dorsal bar; I–VII, hook pairs; pe, penis; va, ventral anchor; vb, ventral bar; and vg, vagina.

**Figure 5:**
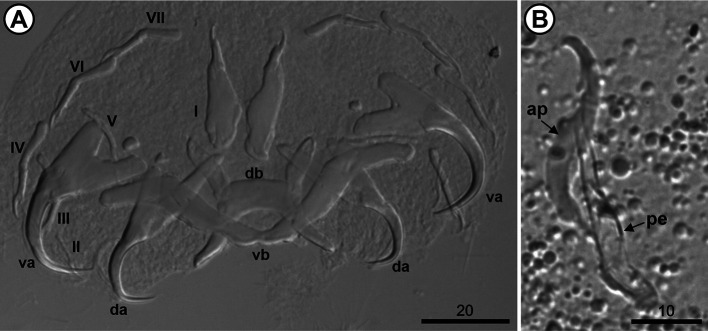
*Cichlidogyrus papernastrema* Price, Peebles & Bamford, 1969 from the gills of *Coptodon rendalli* (Boulenger, 1897) from Pardo River, Paranapanema River basin, São Paulo state, Brazil. A. Haptor; B. Male copulatory complex. Hoyer’s mounting medium. Legend: ap, accessory piece; da, dorsal anchor; db, dorsal bar; I–VII, hook pairs; pe, penis; va, ventral anchor; and vb, ventral bar.

The 10 analyzed *O. niloticus* (mean length: 7.26 [7.4–19.7] cm; mean weight: 10.8 [6.97–164.28] g) were infested with three species of *Cichlidogyrus*, *i.e.*, *Cichlidogyrus mbirizei* Muterezi Bukinga, Vanhove, Van Steenberge & Pariselle, 2012 (Fig. 1), *C. thurstonae* (Fig. 2), and *C. tilapiae* (Fig. 3), and one species of *Scutogyrus,* i.e., *S. longicornis* (Fig. 4). As for the 20 analyzed *C. rendalli* (mean length: 7.18 [3.2–13.0] cm; mean weight: 13.54 [4.5–23.0] g), we only found *Cichlidogyrus papernastrema* Price, Peebles & Bamford, 1969 (Fig. 5). All monogeneans were identified based on the morphology of the reproductive organs and the sclerotized parts of the haptor, which is characterized by baring two pairs of anchors, a V-shaped ventral transversal bar, and a dorsal transversal bar with two auricles and seven pairs of marginal hooks. Measurements of the collected specimens are provided in Table 2.

**Table 2:** Measurements of *Cichlidogyrus* spp. Paperna, 1960 and *Scutogyrus longicornis* (Paperna & Thurston, 1969) collected from *Oreochromis niloticus* (Linnaeus, 1758) and *Coptodon rendalli* (Boulenger, 1897) (Cichlidae) in a river spring in Brazil. Measurements are represented in μm as the average, the range in parentheses, and the count in brackets.

Species	*C. papernastrema*	*C. tilapiae*	*C. mbirizei*	*C. thurstonae*	*S. longicornis*
Host	*C. rendalli*	*O. niloticus*	*O. niloticus*	*O. niloticus*	*O. niloticus*
Number of specimens	*n* = 5	*n* = 10	*n* = 4	*n* = 8	*n* = 1
Total body length	671.2 (495–888) [5]	485.5 (404–557) [10]	567.8 (520–647) [4]	504.4 (315–590) [8]	1238 (1238) [1]
Ventral anchor†					
Total length	32.2 (26–35) [10]	30.5 (28–37) [20]	43.5 (41–45) [8]	31.2 (30–33) [14]	34 (34) [2]
Blade length	27 (26–29) [10]	25.8 (23–30) [20]	39 (36–44) [8]	25.9 (24–27) [14]	33 (33) [2]
Shaft length	9.6 (6–12) [10]	5.7 (5–6) [20]	9.1 (8–11) [8]	8.9 (8–10) [14]	5 (5) [2]
Guard length	12.5 (11–14) [10]	13.8 (10–16) [20]	15.9 (15–17) [8]	13.9 (12–16) [14]	15.5 (14–15) [2]
Point length	10 (7–13) [10]	11.8 (10–13) [20]	17.1 (17–18) [8]	12.5 (12–14) [13]	16 (16) [2]
Dorsal anchor†					
Total length	38.4 (34–42) [10]	37.7 (36–40) [20]	42.3 (39–45) [7]	26.1 (25–29) [14]	31.5 (31–32) [2]
Blade length	22.9 (21–25) [10]	25.3 (23–29) [20]	34.9 (31–40) [7]	20.5 (20–22) [14]	29.5 (28–31) [2]
Shaft length	8.4 (7–10) [10]	4.9 (4–6) [20]	8.6 (8–9) [7] [7]	8.3 (6–10) [14]	8.5 (8–9) [2]
Guard length	17.8 (15–20) [10]	18 (16–20) [20]	16.1 (15–18) [7]	12.3 (11–14) [14]	14 (14) [2]
Point length	8.6 (7–11) [10]	10 (8–11) [20]	14.6 (13–16) [7]	9.7 (8–14) [14]	10 (10) [2]
Ventral bar					
Branch length	45.5 (39–52) [4]	38 (31–46) [10]	45.8 (42–50) [4]	39.1 (28–45) [8]	58 (58) [1]
Maximum width	6.3 (5–7) [4]	7 (6–8) [10]	7.3 (6–8) [4]	5.9 (5–7) [8]	6 (6) [1]
Dorsal bar					
Total length	42.8 (35–49) [5]	38.8 (31–48) [10]	50.5 (43–58) [4]	54.6 (44–61) [8]	53 (53) [1]
Maximum width	6 (5–8) [5]	7.7 (5–9) [10]	9 (8–10) [4]	6 (4–7) [8]	9 (9) [1]
Distance between auricles	18.6 (16–22) [5]	12 (10–15) [10]	24 (22–26) [4]	16.5 (13–20) [8]	28 (28) [1]
Auricle length†	14.4 (13–16) [10]	20.2 (15–25) [20]	18.6 (16–22) [8]	17.8 (15–25) [16]	62.5 (62–63) [2]
Hook†					
Length, I	32.2 (29–37) [10]	15.4 (14–16) [20]	15.1 (13–16) [8]	17.1 (15–19) [14]	20 (20) [2]
Length, II	11.6 (10–15) [9]	11.1 (10–12) [16]	12.8 (11–14) [8]	13.8 (13–14) [8]	11 (11) [2]
Length, III	24.8 (23–27) [10]	17.9 (15–20) [20]	18.4 (16–22) [8]	44.8 (41–51) [12]	36.5 (36–37) [2]
Length, IV	24.3 (21–28) [10]	20.3 (18–22) [20]	22.8 (18–25) [8]	45.4 (43–50) [10]	34 (34) [2]
Length, V	22.1 (19–25) [10]	16.5 (15–18) [20]	18.5 (17–19) [8]	46.2 (40–50) [12]	35 (35) [2]
Length, VI	21.3 (20–22) [8]	17 (14–19) [14]	25.4 (23–28) [8]	45 (38–51) [10]	35.7 (35–36) [2]
Length, VII	16.1 (14–18) [8]	17.8 (14–23) [19]	26.8 (22–28) [8]	45.8 (40–50) [12]	35 (35) [2]
MCO					
Penis length	34.5 (30–38) [4]	–	177.3 (146–200) [4]	41.4 (37–49) [7]	50 (50) [1]
Length of accessory piece	30.5 (24–41) [4]	33.7 (29–38) [6]	55.5 (50–59) [4]	41.1 (37–43) [7]	34 (34) [1]
Heel length	3 (2–4) [3]	–	12.8 (10–15) [4]	5.7 (5–7) [7]	8 (8) [1]
Vagina					
Vaginal length	–	–	–	22.7 (20–26) [7]	32 (32) [1]
Vaginal width	–	–	–	5.9 (5–7) [7]	5 (5) [1]

† Doubled measurements of both structures of the same individual.

*Cichlidogyrus papernastrema* reached the highest prevalence (*P* = 60.0%), although it was found only in *C. rendalli,* followed by *C. thurstonae* (*P* = 40.0%) found in *O. niloticus.* However, greater abundance (*A* = 2.0) and intensity of infestation (MII = 10.0) were observed for *C. tilapiae* found in *O. niloticus.* The ecological parameters of infection of the monogeneans found on the gills of *C. rendalli* and *O. niloticus* are presented in Table 3.

**Table 3:** Ecological data of monogeneans found on the gills of *Coptodon rendalli* (Boulenger, 1897) and *Oreochromis niloticus* (Linnaeus, 1758) in ponds from a river spring in Brazil. *P*%, prevalence as percentage; *A*, mean abundance; MII, mean intensity of infestation.

Host/Monogenean	*P*	*A* (range)	MII (range)
*Coptodon rendalli* (*n* = 20)			
*Cichlidogyrus papernastrema*	60.0	1.7 (0–6)	2.8 (1–6)
*Oreochromis niloticus* (*n* = 10)			
*Cichlidogyrus mbirizei*	10.0	0.4 (0–4)	4
*C. thurstonae*	40.0	1.2 (0–7)	3.0 (1–7)
*C. tilapiae*	20.0	2.0 (0–12)	10.0 (8–12)
*Scutogyrus longicornis*	10.0	0.2 (0–2)	2

### Molecular analyses

We successfully obtained 15 partial sequences of the LSU rDNA gene: nine from specimens of *C. papernastrema* (GenBank accession numbers PP477261–PP477269), four from specimens of *C. tilapiae* (GenBank accession numbers PP477257–PP477260), one from a specimen of *C. thurstonae* (GenBank accession number PP477271), and one from a specimen of *S. longicornis* (GenBank accession number PP477270). Unfortunately, we were not able to obtain good quality sequences from *C. mbirizei*.

**Figure 6:**
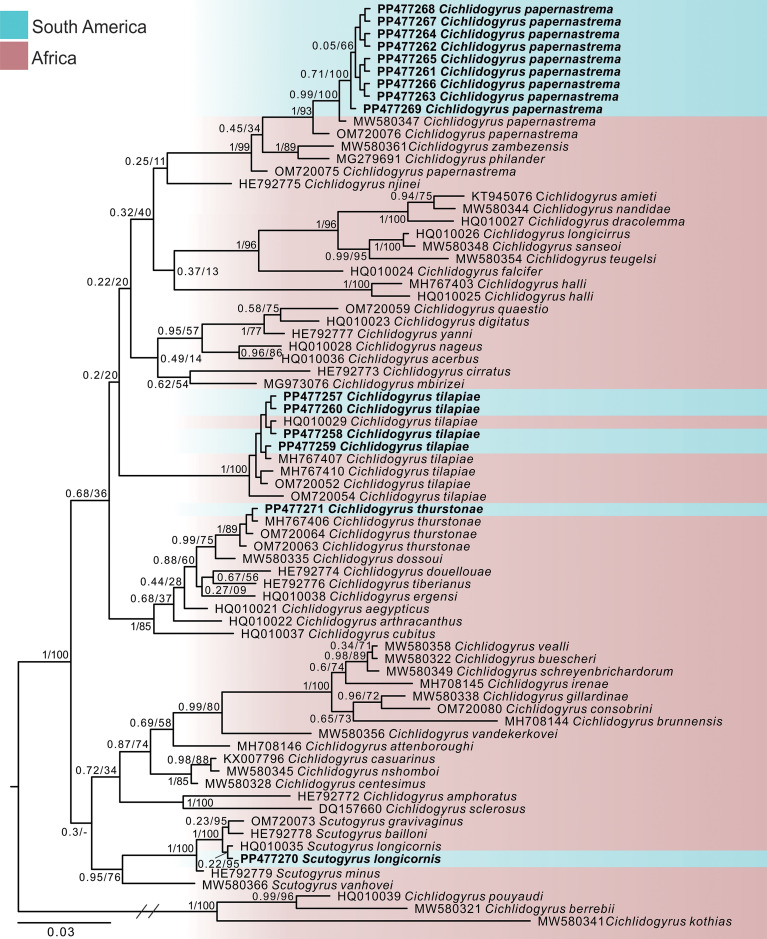
Bayesian topology based on partial LSU rDNA sequences of *Cichlidogyrus* Paperna, 1960 and *Scutogyrus* Pariselle & Euzet, 1995 species. GenBank accession numbers are after species names. Newly sequenced species are in bold. The support values are included above the nodes as follows: posterior probabilities for BI analysis, followed by bootstraps for the ML analysis. Only nodes with posterior probabilities >0.90 and bootstrap scores >70 are considered well-supported. Dashes before nodes represent clades that were not recovered by both analyses. Branch length scale bar indicates the number of substitutions per site.

The final partial LSU rDNA alignment comprised a total of 74 sequences of *Cichlidogyrus* spp. and *Scutogyrus* spp. and, after trimming to the shortest sequence, it was 405 bp long. The BI and ML analyses of the partial LSU rDNA alignment produced phylograms with similar topologies in which the differences were on the level of poorly supported clades, especially on basal branches, and/or unresolved interspecific relationships. The BI topology is shown (Fig. 6), with the BI posterior probabilities (pp) followed by ML bootstrap support values.

Both BI and ML analyses recovered *Cichlidogyrus* spp. as a non-monophyletic group as it included the sequences of *Scutogyrus* spp., which were recovered as monophyletic within *Cichlidogyrus* (pp = 0.95; bootstrap = 76). The newly generated sequences of *C. tilapiae* (pp = 1; bootstrap = 100), *C. thurstonae* (pp = 1; bootstrap = 89), and *S. longicornis* (pp = 0.22; bootstrap = 95) were each grouped together with their conspecific sequences available in the database. Our newly generated sequences of *C. papernastrema* also grouped in a monophyletic clade together with the sequences of *C. papernastrema* (MW580347) collected from *C. rendalli* and *C. papernastrema* (OM720076) collected from *Tilapia sparrmanii* Smith, 1840 (pp = 1; bootstrap = 93) (referred to here as *C. papernastrema “sensu stricto”* clade); however, the sequence of *C. papernastrema* (OM720075) collected from *T. sparrmanii* was placed as the sister taxon to the clade formed by *C. papernastrema “sensu stricto” + Cichlidogyrus zambezensis* Douëllou, 1993 (MW580361) + *Cichlidogyrus philander* Douëllou, 1993 (MG279691) (pp = 1; bootstrap = 99).

Among the sequences of *C. papernastrema “sensu stricto”*, the maximum intraspecific LSU rDNA genetic divergence was 1.4%. The minimum LSU rDNA genetic divergence between the sequence *C. papernastrema* OM720075 and *C. papernastrema “sensu stricto”* was 2.4% (*C. papernastrema* OM720075 × *C. papernastrema* OM720076), while the maximum LSU rDNA genetic divergence was 3.4% (*C. papernastrema* OM720075 × *C. papernastrema* PP477268).

The interspecific LSU rDNA genetic divergences found between the sequence of *C. papernastrema* OM720075 and *C. zambezensis* was 2.4%, whereas the divergence with *C. philander* was 2.0%. In contrast, the interspecific LSU rDNA genetic divergence between *C. papernastrema “sensu stricto”*, and *C. zambezensis* ranged from 3.6% to 4.3%, and concerning *C. philander,* it ranged from 4.0% to 4.7%. The interspecific LSU rDNA genetic divergence between *C. zambezensis* and *C. philander* was 2.1%.

The LSU rDNA genetic divergence within the sequences of *C. papernastrema “sensu stricto”* and the sequences of *C. tilapiae* was 0.3%. No intraspecific genetic variation for the LSU rDNA region was found within the sequences of *C. thurstonae* and *S. longicornis*.

## Discussion

The individuals of *O. niloticus* analyzed in this study were infested with four monogenean species; three of them, *i.e.*, *C. tilapiae*, *C. thurstonae,* and *S. longicornis*, are commonly reported parasitizing the Nile tilapia in Brazil [3, 4, 23, 26, 29, 34, 44, 45, 55, 69, 79]. In contrast, *C. mbirizei* is reported for the first time in the country. *Cichlidogyrus mbirizei* was first described by Muterezi Bukinga *et al*. [53] infesting the gills of *O. tanganicae* (Günther, 1894) in the Democratic Republic of the Congo. Later, the species was found in the Nile tilapia and its hybrids *O. niloticus* × *mossambicus* in Thailand [42], in *O. niloticus* and *Oreochromis* sp. in Malaysia [1, 43] and *Oreochromis mweruensis* Trewavas, 1983 in Africa [35, 75]. In *C. rendalli*, the only species of *Cichlidogyrus* found was *C. papernastrema*, which is also reported for the first time in Brazil. *Cichlidogyrus papernastrema* was first described from *Tilapia sparrmanii* in South Africa [60]. After that, a few recent studies have registered *C. papernastrema* on the gills of *C. rendalli* [14, 31, 33], *T. sparrmanii* [31], and *O. mweruensis* [31, 33], all in the Democratic Republic of Congo. The fact that *C. mbirizei* and *C. papernastrema* have mostly been registered on the African continent reinforces the hypothesis of their co-introduction into Brazil along with their fish hosts brought from Africa, despite some *C. mbirizei* also having been registered in Thailand and Malaysia. In light of that, here we extend the geographical range of two monogenean species, *i.e.*, *C. mbirizei* and *C. papernastrema* and confirm the assessment by Shinn *et al.* [68] regarding the translocation of parasites along with tilapias from their native range in Africa.

Our phylogenetic results agree with previously published analyses on *Cichlidogyrus* spp. and *Scutogyrus* spp., in which *Cichlidogyrus* is non-monophyletic, with *Scutogyrus* forming a monophyletic group nested within it [14, 33, 49, 50, 59, 78]. The representative sequences of *Cichlidogyrus* and *Scutogyrus* used in our analyses formed several supported/unsupported monophyletic groups, showing that the relationships between congeners are still unresolved and require further analyses. Cruz-Laufer *et al*. [14] provided a phylogeny based on a four-locus multiple alignment (partial ITS1, SSU rDNA, LSU rDNA, and COI mtDNA sequence data) along with different comparative methods and parameters (see Cruz-Laufer *et al.* [14] for specific methodology), which recovered similar topology compared to ours albeit with moderate to well-supported clades within *Cichlidogyrus*. This difference was probably generated by the increased set of molecular markers and methods used in those authors’ analyses. It is advisable that future research encompasses datasets with an increased number of DNA sequences from a more complete taxon coverage and molecular markers to provide a detailed picture of the evolution of the genus. Despite that, our phylogenetic results were useful for the correct identification of our *Cichlidogyrus* and *Scutogyrus* specimens, in combination with a detailed morphological analysis. The newly generated sequences are the first from the American continent and the sequences of *C. tilapiae*, *C. thurstonae,* and *S. longicornis* formed reciprocally monophyletic clades with their correspondent conspecific sequences available in the GenBank database from other localities. Interestingly, our molecular results recovered sequences of *C. papernastrema* as a non-monophyletic assemblage, casting doubts on the correct identification of some of the sequences deposited in GenBank; this requires further verification (see “Results” section; Fig. 6).

The maximum intraspecific LSU rDNA genetic divergence found among our sequences of *C. papernastrema “sensu stricto”* was 1.4%, which is in agreement with the maximum intraspecific LSU rDNA distance values found among specimens of *Cichlidogyrus* spp. (1.4%) shown by Jorissen *et al*. [33]*.* Our results also demonstrated that the minimum interspecific genetic distance between the sequences of *C. papernastrema “sensu stricto”* and *C. papernastrema* OM720075 (2.4%) is larger than the genetic distance found between *C. zambezensis* and *C. philander* (2.1%). Therefore, it is possible that the specimens from which the sequences of *C. papernastrema “sensu stricto”* were generated and the specimen from which the sequence *C. papernastrema* OM720075 was obtained might represent separate species. This hypothesis, previously suggested by Jorissen *et al*. [33], is confirmed here considering both our phylogenetic trees and genetic distances.

The morphological measurements from our *C. papernastrema* specimens are congruent with the data presented by Price [60] for the holotype of *C. papernastrema* collected in *T. sparrmanii* from South Africa, and by Jorissen *et al*. [32] for *C. papernastrema* collected in *T. sparrmanni* and *C. rendalli* from the Democratic Republic of Congo (Supplementary Table 1). Therefore, we propose that all these specimens are conspecific. Nevertheless, Jorissen *et al*. [32] noted that the *C. papernastrema* specimens collected in *O. mweruensis* from the Democratic Republic of Congo presented some morphological differences mostly related to the size of anchors and bars and the total length of the body, which the authors considered as intraspecific variation possibly related to host specificity. Future studies should include sequences of *C. papernastrema* collected from different host localities to test this hypothesis. Moreover, Jorissen *et al*. [31, 32] noted a large variation in the thickness of the copulatory tube in specimens of *C. papernastrema*, suggesting that such variation should be further investigated as a possible diagnostic character to delineate species along with genetic analyses [33].

Furthermore, several studies addressing monogenean parasites of *C. rendalli* and *O. niloticus* in Brazil have reported unidentified *Cichlidogyrus* sp. [3, 23, 26, 44, 45, 63, 73, 79] which could not be identified based on the morphology. Difficulties related to feasible and/or correct identification of monogeneans, such as the minute size of the parasites and/or inefficient collection and preservation methods, could be compromising the specific identification of those specimens since these monogeneans are predominantly diagnosed based on the morphology of their sclerotized parts from the reproductive organs and the attachment organ (haptor). Therefore, genetic information is very useful to confirm their taxonomic status. We propose that many *Cichlidogyrus* sp. found in previous studies could now be correctly identified at the species level, especially with the aid of molecular approaches. This is the case of *C. papernastrema* and *C. mbirizei*, which are added here to the bulk list of *Cichlidogyrus* species found in Brazil. Also, the fact that only one monogenean species, *i.e.*, *C. papernastrema*, was found infesting *C. rendalli*, which is in contrast with the scarce literature on parasites of this cichlid species in Brazil [26, 73], highlights that possible misidentifications on both hosts and parasites might still represent a challenge yet to be overcome regarding parasitological studies in Brazil. The addition of new molecular data is still highly appreciated and contributes substantially to the phylogenetic interrelationships of the group.

The tilapias *C. rendalli* and *O. niloticus* have been translocated around the world, including to Brazil, for different purposes [11, 14, 68]. Despite the benefits to human society, tilapia aquaculture and open-water introductions can be very prejudicial to the native environment [11], since these fishes are highly invasive species and exist under feral conditions. The tilapias *C. rendalli* and *O. niloticus* may disrupt ecological native fish communities by competing and feeding on their resources, maintaining a strong territorial behaviour with multiple and expanded spawning, and presenting strong parental care [2, 5, 11, 57]. Moreover, as fishes with great food plasticity and omnivorous feeding habits, *C. rendalli* and *O. niloticus* can feed on zooplankton, phytoplankton, detritus, sediments, insect larvae, eggs, and even young fish of native species [2]. This can be extremely problematic when it comes to an area near a river spring, as is the case of our study. River springs are known as important breeding sites and refuges for several species of native biota (fishes, insects, snails, etc.), especially in the early stages of their development. The presence of *C. rendalli* and *O. niloticus* in an area of a river spring can adversely impact ecosystem services and cause a drastic decline in native biodiversity. In fact, many studies have already demonstrated the negative impact of the introduction of tilapias on the native fauna [2, 5, 11, 61]. For example, the invasion of *Oreochromis* sp. in lakes of Nicaragua reduced greatly the native cichlid populations due to environmental competition [47]. In Panama, the introduction of *O. niloticus* led to the extinction of two endemic species of cichlids, due to several factors including diseases and loss of habitat [65]. Lastly, in Brazilian public reservoirs, the dominance of the Nile tilapia *O. niloticus* has drastically altered the composition of the native fish populations, jeopardizing local artisanal fishing [2, 54].

Another serious risk to native freshwater fishes is the introduction of exotic fishes such as *C. rendalli* and *O. niloticus* which may also introduce alien parasites associated with their hosts [63, 68]. The problem with alien parasites is that they might switch hosts and successfully infect native species [24], increasing the risk of extinction of native fauna. Tilapia parasite host-switching events to native hosts (spillover effect) have already been reported for cichlid, poeciliid, and goodeid fishes in Mexico (*Cichlidogyrus*, *Gyrodactylus* and *Enterogyrus* species) [19, 20, 30, 67, 71], for cichlid fishes from America (*C. sclerosus*, *C. tilapiae*, *S. longicornis*, and *Enterogyrus malmbergi* Bilong Bilong, 1988) [30, 67] and aplocheilid fishes from Madagascar (*C. tilapiae*) [71]. There is also evidence of native parasite fauna infecting introduced tilapias *Oreochromis mossambicus* (Peters, 1852) in Colombia [40]. Moreover, there are documented host-switching events of dactylogyrids from the Nile tilapia being transferred towards native tilapias on their native continent [22]. Although the transmission of monogenean parasites to new hosts is poorly reported, it is evident that dactylogyrids that manage to establish outside their native range can exploit a phylogenetically broad host range [68].

The scenario presented here regarding the introduction of tilapias into wild environments and the potential spillover of their parasites may cause further damage to native fish species and biodiversity in the studied area, unless the growth of tilapia populations is controlled. We strongly recommend additional ecological and parasitological studies to better understand the role of tilapia and their parasites. There is also an imminent need for the implementation of a management plan to control this alien species, to prevent the extinction of native species in an area of a river spring due to horizontal transmission events among fishes.

## Supplementary material

The Supplementary materials of this article are available at https://www.parasite-journal.org/10.1051/parasite/2024021/olm. *Supplementary Table 1*: Measurements of *Cichlidogyrus papernastrema* Price, Peebles & Bamford, 1969. Measurements are represented in μm as the average, the range in parentheses, and the count in brackets († = doubled measurements of both structures of the same individual). * = Holotype.
