# Supplementary material for: Parasites (Monogenea) of tilapias Oreochromis niloticus and Coptodon rendalli (Cichlidae) in a river spring in Brazil
Source: Parasite. 2024 Apr 10;31:22. doi: 10.1051/parasite/2024021 (PMC11008226; doi:10.1051/parasite/2024021)
Supplement: Supplementary file 1 — Supplementary Table 1: Measurements of Cichlidogyrus papernastrema Price, Peebles & Bamford, 1969. Measurements are represented in μm as the average, the range in parentheses, and the count in brackets († = doubled measurements of both structures of the same individual). * = Holotype. [file parasite-31-22-s1.pdf]

Supplementary Table 1. Measurements of *Cichlidogyrus papernastrema* Price, Peebles & Bamford, 1969. Measurements are represented in µm as the average, the range in parentheses, and the count in brackets († = doubled measurements of both structures of the same individual). * = Holotype.

	C. papernastrema				
**Host**	*T. sparrmanii*	*C. rendalli*	*C. rendalli*	*O. mweruensis*	*T. sparrmanii*
**Locality**	South Africa	Brazil	DR Congo	DR Congo	DR Congo
**Reference**	Price et al., 1969	This study	Jorissen et al., 2018	Jorissen et al., 2018	Jorissen et al., 2018
**Number of specimens**	n = 1*	n = 5	n = 5	n = 5	n = 10
**Total body length**	273	671.2 (495-888) [5]	381 (190–473) [5]	254 (240–272) [4]	385 (270–631) [5]
**Ventral anchor**†					
Total length	33	32.2 (26-35) [10]	31 (29-34) [4]	26 (25-27) [2]	32 (31-32) [3]
Blade length	29	27 (26-29) [10]	26 (24-28) [4]	23 (21-24) [2]	27 (24-30) [3]
Shaft length	8	9.6 (6-12) [10]	6 (5-6) [3]	3 (3-3) [2]	5 (4-6) [3]
Guard length	13	12.5 (11-14) [10]	13 (12-14) [4]	7 (7-7) [2]	12 (12-13) [3]
Point length	10	10 (7-13) [10]	11 (8-13) [4]	10 (9-10) [2]	12 (11-12) [3]
**Dorsal anchor**†					
Total length	38	38.4 (34-42) [10]	37 (31-40) [4]	28 (28-28) [2]	38 (33-44) [2]
Blade length	25	22.9 (21-25) [10]	25 (24-28) [4]	16 (13-19) [2]	24 (20-29) [2]
Shaft length	7	8.4 (7-10) [10]	6 (6-7) [4]	5 (3-6) [2]	6 (5-7) [2]
Guard length	17	17.8 (15-20) [10]	17 (15-19) [4]	14 (12-16) [2]	18 (16-20) [2]
Point length	8	8.6 (7-11) [10]	10 (8-12) [2]	8 (7-10) [2]	9 (9-10) [2]
**Ventral bar**					
Branch length	45	45.5 (39-52) [4]	39 (30-45) [3]	30 (28-32) [2]	42 (37-51) [5]
Maximum width	6	6.3 (5-7) [4]	6 (4-8) [3]	4 (4-4) [2]	6 (4-7) [5]
**Dorsal bar**					
Total length	32	42.8 (35-49) [5]	43 (38-48) [5]	26 (26-26) [2]	41 (35-52) [4]
Maximum width	7	6 (5-8) [5]	7 (7-8) [5]	7 (5-9) [2]	8 (7-10) [4]

Distance between auricles	11	18.6 (16-22) [5]	15 (12–18) [5]	9 (9-10) [3]	15 (13–18) [4]
Auricle length†	18	14.4 (13-16) [10]	17 (16–20) [10]	11 (10–13) [2]	16 (12–19) [4]
**Hooks**†					
Length, I	28	32.2 (29-37) [10]	28 (22–33) [5]	24 (24–25) [2]	30 (27–36) [5]
Length, II	12	11.6 (10-15) [9]	10	13	11
Length, III	21	24.8 (23-27) [10]	17 (17–17) [3]	19 (19–20) [2]	17 (15–19) [2]
Length, IV	21	24.3 (21-28) [10]	23 (21–25) [4]	19	23 (21–25) [2]
Length, V	23	22.1 (19-25) [10]	23 (17–28) [4]	20 (19–22) [2]	29 (27–30) [2]
Length, VI	20	21.3 (20-22) [8]	24 (19–29) [2]	20 (20–32) [2]	28 (24–33) [2]
Length, VII	16	16.1 (14-18) [8]	19 (14–22) [4]	18 (18–19) [2]	25 (24–27) [2]
**MCO**					
Penis length	32	34.5 (30-38) [4]	32 (26-44) [10]	30 (28-36) [5]	26 (23-30) [3]
Length of accessory piece	37	30.5 (24-41) [4]	42 (33-47) [10]	37 (33-41) [4]	30 (24-40) [3]
Heel length	1	3 (2-4) [3]	2 (1-3) [9]	2 (2-2) [4]	3 (3-4) [2]
